# Current Review of the Function and Regulation of Tuberoinfundibular Dopamine Neurons

**DOI:** 10.3390/ijms25010110

**Published:** 2023-12-21

**Authors:** Xiaojun Qi-Lytle, Sarah Sayers, Edward J. Wagner

**Affiliations:** 1 Department of Medical Education, Geisinger Commonwealth School of Medicine, 525 Pine St., Scranton, PA 18509, USA; xqilytle@geisinger.edu; 2 Department of Basic Medical Science, College of Osteopathic Medicine of the Pacific, Western University of Health Sciences, 309 E. Second St., Pomona, CA 91766, USA; sarah.sayers@westernu.edu

**Keywords:** TIDA neurons, dopamine, prolactin, thyrotropin releasing hormone, estradiol, glutamate, GABA, opioids, lactation

## Abstract

Tuberoinfundibular dopamine (TIDA) neurons have cell bodies located in the arcuate nucleus of the mediobasal hypothalamus. They project to the external zone of the median eminence, and the dopamine (DA) released there is carried by the hypophysial portal vasculature to the anterior pituitary. The DA then activates D2 receptors to inhibit prolactin (PRL) secretion from lactotrophs. The TIDA neuronal population is the principal regulatory factor controlling PRL secretion. The neuroendocrine role subserved by TIDA neurons sets them apart from other dopaminergic populations like the nigrostriatal and mesolimbic DA neurons. TIDA neurons exhibit intrinsic oscillatory fluctuations in their membrane potential that give rise to phasic firing and bursting activity. TIDA neuronal activity is sexually differentiated and modulated by gonadal hormones and PRL, as well as an array of small molecule and peptide neurotransmitters. This review covers these characteristics.

## 1. Common Features of Dopaminergic Neurons

### 1.1. Dopaminergic Neurons

**Figure 1:**
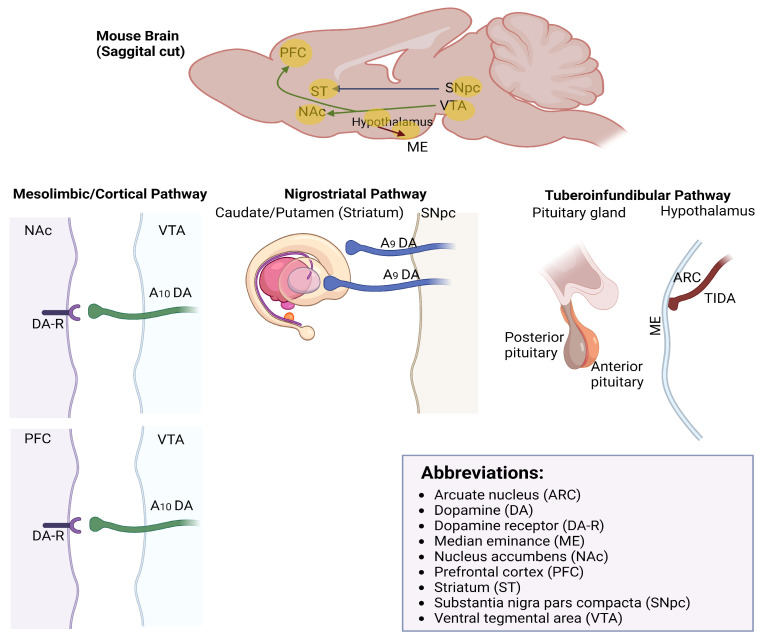
Anatomical organization of the nigrostriatal (A9), mesolimbic/cortical (A10), and tuberoinfundibular DA pathways.

Dopaminergic neurons are specialized nerve cells that produce and release the neurotransmitter DA. These neurons are primarily located in specific regions of the brain, including the substantia nigra (nigrostriatal DA neurons), ventral tegmental area (mesolimbic/mesocortical DA neurons), and hypothalamus (Figure 1). The nigrostriatal and mesolimbic/mesocortical DA neurons play a crucial role in various aspects of brain function, including reward, motivation, movement, mood regulation, and cognitive processes [1]. Dopaminergic neurons often exhibit specific firing patterns, characterized by phasic (bursting) and tonic (regular) activity. Phasic firing is associated with the rapid release of DA and is implicated in reward-related processes, while tonic firing maintains baseline DA levels necessary for ongoing neurotransmission [2]. Like other neurons, dopaminergic neurons can exhibit plasticity, allowing them to adapt their firing patterns and synaptic connections in response to experiences and learning. This plasticity is essential for encoding reward-related information and responding to changing environmental cues [3].

### 1.2. Dopaminergic Receptors

Dopaminergic receptors are classified into two categories based on their location: autoreceptors, found on the same neuron that released DA, and heteroreceptors, located on neighboring neurons or target cells. These receptors play a crucial role in regulating dopaminergic neuron activity through feedback mechanisms and can also influence the activity of neighboring neurons. Additionally, DA receptors are all metabotropic G-protein-coupled receptors with seven transmembrane domains arranged in serpentine fashion, with a large extracellular N terminus and intracellular C terminus. They can be further categorized into two families: D1-like receptors (D1 and D5), which predominantly activate the Gs signaling pathway, leading to increased intracellular cyclic AMP levels, and D2-like receptors (D2, D3, and D4), which primarily inhibit adenylyl cyclase, activate G-protein-gated inwardly rectifying potassium channels and inhibit voltage-gated calcium channels through the Gi signaling pathway. These receptor distinctions help fine-tune the effects of DA signaling in various brain regions and physiological processes [4,5].

### 1.3. DA Synthesis

DA synthesis is a multi-step process that begins with tyrosine, an amino acid, which serves as the precursor to DA. The rate-limiting enzyme tyrosine hydroxylase (TH) converts tyrosine to L-3,4-dihydroxyphenylalanine within the cytoplasm of dopaminergic neurons. L-Dopa is further converted to DA by the enzyme aromatic L-amino acid decarboxylase. This enzyme removes a carboxyl group from L-Dopa, resulting in the formation of DA. This conversion takes place in the cytoplasm as well. Once synthesized, DA is transported into synaptic vesicles by the vesicular monoamine transporter. When an action potential reaches the dopaminergic neuron’s axon terminal, it triggers the release of DA from the vesicles into the synaptic cleft. DA then binds to its receptors on postsynaptic neurons, mediating various physiological effects [6].

### 1.4. DA Metabolism

DA can also be metabolized and degraded by enzymes. These activities ensure that excess DA is broken down and cleared from the synapse. The primary enzymes responsible for the degradation of DA are monoamine oxidase (MAO) and catechol-O-methyltransferase (COMT). MAO—specifically, MAO-B—is located on the outer mitochondrial membrane of dopaminergic neurons. This enzyme plays a crucial role in the breakdown of excess DA within the neuron. MAO oxidizes DA into a metabolite known as dihydroxyphenylacetic acid (DOPAC). COMT is primarily found in glial cells and postsynaptic neurons. COMT methylates DA, converting it into homovanillic acid, another metabolite. This process occurs both within the synapse and in the extracellular fluid [6].

### 1.5. DA Negative Feedback

**Figure 2:**
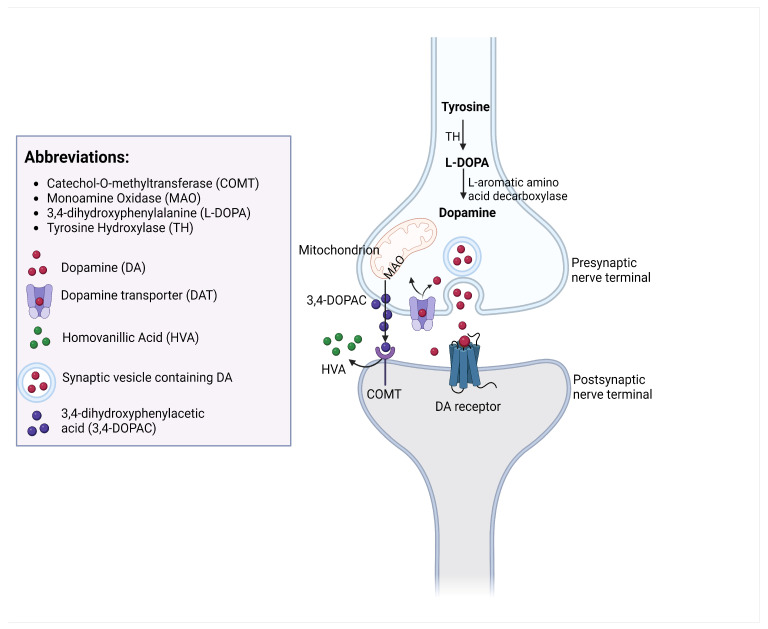
The processes of DA synthesis, reuptake, and metabolism occurring at a dopaminergic synapse.

DA autoreceptors are situated on dopaminergic neurons and are activated by DA itself. These autoreceptors play a pivotal role in regulating the dopaminergic system by providing feedback inhibition that governs cell firing, as well as the synthesis, release, and reuptake of DA [7]. All DA autoreceptors belong to the D2 receptor family and, among these, the D2 receptor subtype are well-established as the primary autoreceptors [8]. The receptors are like sensors on the dopaminergic neurons that detect DA levels. When DA levels in the synapse get too high, the D2 receptors are activated upon ligand binding. This activation sets off a signal within the neuron that tells it to make and release less DA. After DA is released into synapses, it can be taken back up by the axon of the neuron. DA reuptake is facilitated by specialized DA transport (DATs) proteins. These DATs are primarily located on the presynaptic membrane of dopaminergic neurons, adjacent to the synapses [9]. DATs effectively pump DA from the synapse and return it to the neuron for repackaging and reuse in future signaling events. The reuptake of DA terminates the signaling effects of DA at the synapse and helps control the duration and intensity of DA signaling by retrieving excess DA from the synapses, which allows for precise control of neurotransmission, preventing both excessive stimulation and depletion. This regulation mechanism has been used as the target for many psychiatric medications. The variations in the function or density of DA reuptake transporters can contribute to individual differences in DA signaling. It may influence susceptibility to psychiatric conditions or responsiveness to medications that target the DA system [9]. The processes of DA synthesis, reuptake, and metabolism are schematized in Figure 2.

## 2. TIDA Neurons

1. Anatomical Structure

The dopaminergic pathways in the brain originate from various regions, including the substantia nigra pars compacta, ventral tegmental area, and hypothalamus. Each of these pathways project to specific areas to play distinct functions. While these pathways often collaborate with other neurons, the tuberoinfundibular dopaminergic pathway stands out as a unique neural and endocrine connection. The tuberoinfundibular pathway initiates its course in the arcuate nucleus of the hypothalamus at the brain’s base. The neurons have a characteristic shape with a mean length-to-width profile of approximately 14.9 × 11.5 microns [10]. The DA is released from TIDA neurons in the external zone of the median eminence (ME) and subsequently transported by the hypophysial portal blood vasculature to the anterior pituitary gland, where it exerts a potent suppressive influence on pituitary lactotroph cells to tonically inhibit PRL secretion [11].

2. Functions

In contrast, the nigrostriatal, mesocortical, and mesolimbic pathways primarily govern central nervous system functions such as motor control, reward processing, and mood regulation. The TIDA pathway is a critical component of the neuroendocrine system responsible for maintaining hormonal balance and regulating reproductive and metabolic functions in the body. It illustrates the intricate communication between the brain and the endocrine system to ensure proper physiological control. TIDA targets the lactotroph cells in the anterior pituitary to tonically inhibit the secretion of PRL [12,13,14,15]. PRL is involved in various physiological processes, with its most well-known role being the stimulation of milk production in the mammary glands. Not surprisingly, D2 agonists like bromocriptine are used to treat hyperprolactinemia seen in various types of amenorrhea.

TIDA neurons exert their inhibitory control over PRL secretion through a feedback mechanism. When PRL levels increase, they stimulate DA release from the TIDA neuron to inhibit PRL synthesis. Consequently, the secretion of PRL is reduced or suppressed, preventing excessive levels of the hormone. However, during stress and lactation, the excitability of TIDA neurons is decreased, thus allowing for heightened PRL secretion triggered by the suckling reflex [16,17,18].

3. Inherent Membrane Characteristics

Most midbrain DA neurons share a common feature of spontaneous firing action without requiring external stimuli [19,20]. TIDA neurons also exhibit this pacemaker-like firing pattern [21]. The presence of small-conductance, calcium-activated potassium channels of subtype 3 (SK3) channels, coupled with T-type calcium channels underlying the low-threshold spike capable of generating bursts of action potentials, play a critical role in governing the rhythmic and pacemaker-like firing behaviors seen in DA neurons [10,22,23]. Additionally, TIDA neurons typically have a membrane potential that exhibits intermittent depolarizing fluctuations of 30 mV in amplitude lasting many seconds. These fluctuations allow for oscillatory firing patterns characterized by rhythmic bursts of action potentials. This oscillatory behavior leads to the pulsatile release of DA, and what is remarkable is that this robust oscillation is synchronized among multiple TIDA neurons within the same network or region. This synchronization is highly dependent on intact gap junction communication, which facilitates direct electrical and chemical communication between neighboring TIDA neurons [11,24]. These intrinsic characteristics, including spontaneous firing, depolarized resting membrane potential, and synchronized oscillatory firing, collectively define TIDA neurons. They serve as unique features of inhibiting PRL secretion by providing continuous tonic inhibition through the release of DA.

4. Regulation of TIDA Neurons

The regulation of TIDA neurons is a highly intricate and interconnected process involving multiple neurotransmitters, neuropeptides, and hormones. The exact mechanisms and interactions can vary depending on the receptor subtypes involved. We will now focus on these regulatory inputs.

### 2.1. Regulation by Autoreceptors

The activity of TIDA neurons can be influenced by various neurotransmitters and neuromodulators, including DA itself through the autoreceptors. DA autoreceptors, primarily of the D2 subtype, were identified on the soma, dendrites, and axon terminals of midbrain DA neurons, notably in regions like the ventral tegmental area and substantia nigra pars compacta. These autoreceptors play a pivotal role in regulating DA by modulating synthesis, release, and impulse traffic, depending on their locations on the neuron [25,26,27,28]. However, it is worth noting that the autoreceptors in TIDA neurons exhibit unique characteristics that have sparked ongoing debates. In contrast to dopaminergic neurons in the ventral tegmental area and substantia nigra pars compacta, the existence of autoreceptors directly influencing DA synthesis through modulation was not initially well-established in TIDA neurons [29]. Over the subsequent years, research groups embarked on a quest to definitively identify D2 autoreceptors on TIDA neurons, but conclusive evidence remained elusive. For instance, a study by Anden et al. in 1983 demonstrated that the regulation of DA by DA autoreceptor agonists or antagonists was observed in the corpus striatum, the nucleus accumbens, the olfactory tubercle, and the limbic cortex, but not in TIDA neurons [30,31]. In 1995, Timmerman et al. similarly reported a lack of evidence for the existence of autoreceptors in TIDA neurons based on using the selective D2 antagonist sulpiride [32].

In 2016, Stagkourakis’ research made significant progress in this area by investigating changes in TIDA neuron electrical behavior in response to ambient somatodendritic DA concentration. Their study identified D2 autoreceptors on the soma of TIDA neurons. Activation of these receptors was found to reduce TIDA neuron oscillation frequency, while inhibition of these receptors led to depolarization block. Furthermore, DATs located on TIDA neuron somas were shown to influence cell oscillation by reuptaking ambient somatodendritic DA. In sum, Stagkourakis’ research highlighted the existence of a unique ultra-short feedback loop in the neuroendocrine TIDA neurons: DA levels in specific cellular regions adjusted network oscillations through a combination of pre- and postsynaptic actions, ultimately regulating DA release [33]. While direct pre-synaptic regulation of DA synthesis by D2 receptors in TIDA neurons may not be well-supported [29], it is possible that pre-synaptic DATs indirectly impact DA synthesis. Increased intracellular DA levels, resulting from reuptake via DATs, can inhibit the activity of tyrosine hydroxylase, a rate-limiting enzyme in DA synthesis, ultimately leading to a decrease in DA production.

### 2.2. Regulation by PRL, Estradiol, and Testosterone

PRL release is typically inhibited by TIDA neurons; however, when these neurons are exposed to high concentrations of PRL, it triggers an increase in DA synthesis and release. Gonzalez et al. revealed that PRL enhances the enzymatic activity of TH, leading to greater DA production. Remarkably, this effect is not associated with changes in the amount of TH or alterations in TH phosphorylation [34]. The increase in DA release from TIDA neurons induced by PRL operates through a feedback mechanism. Elevated serum PRL levels simultaneously boost the firing rate and duration of TIDA neurons, causing them to transition from phasic (intermittent) to tonic (continuous) discharge patterns [35]. This, in turn, facilitates DA release, which serves as a regulator to curb further PRL secretion. This discovery sheds light on a novel feedback mechanism that plays a significant role in regulating PRL secretion and, consequently, impacts various aspects of reproductive and metabolic physiology.

Estradiol exhibits a complex regulatory role in the control of PRL levels, with both stimulatory and inhibitory effects. For instance, 17 beta-estradiol can increase PRL production by regulating PRL gene transcription, possibly through various mechanisms that are independent of pituitary proteins [36,37]. However, this rise in PRL levels subsequently stimulates TIDA neurons to release more DA, which, in turn, acts as an inhibitor of PRL secretion [38]. This intricate interplay involving estradiol ensures a delicate balance between its ability to stimulate PRL synthesis and its influence on DA-mediated inhibition of PRL secretion. Moreover, 17 beta-estradiol has the capacity to directly modulate the excitability of TIDA neurons, depending on the specific hormonal and physiological context in which estradiol operates. For example, 17 beta-estradiol has been observed to enhance the expression of small-conductance SK3 and T-type calcium channels. Notably, estradiol treatment leads to an increase in SK3 mRNA levels, particularly in the rostral basal hypothalamus, especially during the negative feedback phase of the reproductive cycle. SK3 activation contributes to hyperpolarization during the afterhyperpolarization (AHP) phase, thus exerting an inhibitory effect on cell excitability [23]. In contrast, T-type calcium channels, also known as low-threshold calcium channels, play a distinct role in influencing a neuron’s firing pattern by generating low-threshold spikes and facilitating burst firing [39]. This complex interaction between estradiol, PRL, and neuronal excitability underscores the multifaceted nature of hormonal regulation in this neuroendocrine system.

Estradiol’s pivotal role in regulating TIDA neurons is consistent with sexual differences in TIDA neuronal activity. The basal activity of TIDA neurons is higher in females than in males. This difference is eliminated upon gonadectomy and restored by gonadal hormone replacement [40,41,42,43,44]. As discussed above, estradiol indirectly stimulates TIDA neurons by stimulating PRL synthesis and secretion [38]. Conversely, testosterone inhibits their activity [43]. However, testosterone does not alter the ability of TIDA neurons to respond to prolactin feedback, which distinguishes its impact from estradiol [38,44]. This sexual difference extends to the functional role of TIDA neurons. As discussed below, TIDA neurons are inhibited by suckling in lactating female rats—a situation that does not present itself in male subjects. These neurons also respond to stress in a sexually differentiated way. For example, while stress robustly increases PRL secretion, this is accompanied by decreased TIDA neuronal activity in female and orchidectomized male rats but not in intact or orchidectomized, testosterone-treated male rats [43,44].

### 2.3. Regulation by Glutamate

Glutamate is the major excitatory neurotransmitter in the nervous system. In the early 1990s, many glutamate receptor subunits were cloned, including those comprising the N-methyl-D-aspartate (NMDA) receptors. When NMDA receptors are activated, they allow calcium and sodium ions into neurons, leading to neuronal excitation [45,46]. In 1991, Arslan et al. conducted a study using adult rats to explore the involvement of excitatory amino acids, specifically those that activate the NMDA receptor, in regulating PRL secretion. The experiment involved the administration of NMDA receptor agonists and antagonists, with subsequent measurements of PRL and LH levels. The results clearly demonstrated that active NMDA receptors led to an increase in LH levels. However, the findings regarding PRL secretion were less conclusive. Both agonists and antagonists of the NMDA receptor appeared to elevate PRL secretion. This suggests that the regulation of PRL through the NMDA receptor may be influenced by various factors, highlighting the complexity of this regulatory mechanism [47]. A few years later, Wagner et al. conducted further studies that provided additional insights into the regulation of TIDA neurons through NMDA receptors. Their research revealed that NMDA receptor antagonists had distinct effects on TIDA neurons in male and female rats. NMDA receptor antagonism significantly decreased DA turnover in the ME of female but not male subjects. Additionally, they made a significant discovery that estradiol positively modulates the NMDA receptor-mediated tonic stimulation of TIDA neurons in female rats through a mechanism that is independent of PRL [40].

While continuing their investigation, Wagner et al. turned their attention to the alpha-amino-3-hydroxy-5-methylisoxazole-4-propionic acid (AMPA) receptors, a distinct type of excitatory glutamate receptor. In contrast to NMDA receptors, AMPA receptors are primarily permeable to sodium and potassium ions, leading to a rapid influx of sodium ions and subsequent postsynaptic membrane depolarization. These receptors play a central role in mediating fast excitatory neurotransmission within the brain. Their study revealed that the blockade of AMPA receptors resulted in an increase in 3,4-DOPAC concentrations specifically within the ME and intermediate lobe of the pituitary gland, but not in the striatum. Furthermore, this blockade led to a dose- and time-dependent reduction in both PRL and alpha-melanocyte-stimulating hormone levels. These findings shed light on the regulatory mechanisms governing dopaminergic neurons in relation to non-NMDA receptor activity. They highlight the role of endogenous excitatory amino acid neurotransmitters, acting at AMPA receptors, in tonically inhibiting TIDA neurons, ultimately resulting in increased secretion of PRL in both male and female rats [48]. The precise mechanism behind how the activation of excitatory glutamate receptors on TIDA neurons leads to decrease DA secretion remains unclear, although follow-up research indicates that this AMPA receptor-mediated tonic inhibition of TIDA neurons occurs through intervening GABAergic neurons [49]. Commonly, cognitive processes involve the cross-talking between glutamate and gamma-aminobutyric acid (GABA) through their receptors, which include NMDA and AMPA receptors [50]. Activation of these receptors may facilitate GABAergic inhibitory input.

### 2.4. Regulation by GABA

GABA exerts an inhibitory influence on the TIDA system under normal physiological conditions. This regulation helps maintain appropriate levels of PRL secretion, particularly in individuals who are not lactating. TIDA neurons are known to co-express GABA, indicating that they release GABA as well [51]. Released GABA can act as a neurotransmitter within the arcuate nucleus to influence local circuits [52]. To understand the mechanisms by which GABA regulates TIDA neurons and, consequently, PRL levels, various research teams have used GABA receptor agonists and antagonists. Their investigations have led to consistent conclusions regarding GABAA receptors. Activation of GABAA receptors has been found to suppress the release of DA in the ME, leading to an increase in pituitary PRL levels. Conversely, blocking GABAA receptors reverses this suppression [53,54,55].

Regarding GABAB receptor activation, some studies indicated that administration of the GABAB receptor agonist, baclofen, either peripherally or centrally, did not significantly impact DOPAC levels in the ME or serum PRL levels [53,54]. Other studies observed that baclofen indeed inhibited TIDA neurons, thus reducing DOPAC levels and robustly increasing plasma PRL levels. When a GABAB receptor antagonist, 2-Hydroxysaclofen, was pre-administered, it blocked the baclofen-induced changes, supporting the notion that GABAB receptors play an appreciable role in this context [55]. In 2022, Ammari and colleagues contributed to our understanding of GABAB receptor regulation. They found that GABAB receptors act as a brake on TIDA neurons, potentially serving as auto/heteroreceptors that provide inhibitory feedback. GABAB receptor activation has a dual role in modulating TIDA neurons. Postsynaptically, it leads to the opening of potassium-like channels, resulting in outward currents and hyperpolarization in TIDA neurons. Presynaptically, GABAB receptors limit the release of neurotransmitters (glutamate and GABA) onto TIDA neurons. This means that GABAB receptors not only affect the electrical properties of TIDA neurons but also regulate the signals they receive from other neurons [56]. Along similar lines, and as will be discussed below, the thyrotropin-releasing hormone (TRH) works as a counter to this GABAB-receptor-mediated presynaptic inhibition by enhancing GABA and glutamate input onto TIDA neurons. Overall, these findings highlight the intricate interplay between amino acid neurotransmitters and TIDA neurons in the control of PRL secretion, shedding light on the complex mechanisms involved in maintaining hormonal balance in the body.

### 2.5. Regulation by Neuropeptides

#### 2.5.1. TRH

TRH has been shown to increase PRL secretion by directly influencing lactotrophs [57]. However, TRH receptors have also been identified on TIDA neurons, suggesting the existence of a direct TRH–TIDA neuron interaction. This notion is supported by various research articles. For example, one study conducted in 1985 used quantitative autoradiography to map the distribution of TRH receptors in the rat brain. This study revealed varying concentrations of TRH receptors in different brain regions, with moderate concentrations found in the hypothalamus, including the arcuate nucleus and ME [58]. In another study, which utilized the technique of in situ hybridization histochemistry, researchers identified a specific subtype of TRH-R1 mRNA that was predominantly expressed in hypothalamic regions, including the arcuate nucleus [59]. Additionally, TRH neurons make synaptic contact with TIDA neurons [60]. TRH binding sites are found in TIDA neurons, and TRH potently and robustly increases DA release and intracellular calcium concentrations in these cells [61]. Further, central administration of TRH increases DA turnover in the hypothalamus and the ME, and decreases PRL secretion [62,63]. The mechanism by which TRH regulates TIDA neuron activity was more recently investigated by Lyons et al. in 2010, providing new insights into this process. Their research proposed a model in which TRH exerts its effects on TIDA neurons through both pre- and postsynaptic influences. TRH elicits an inward current caused by activation of a mixed cation channel that depolarizes the membrane potential of TIDA neurons and induces a transition from phasic firing to tonic firing patterns. Additionally, abolishment of spontaneous bursting may disrupt synchronization within the TIDA neuron population and possibly compromise the efficiency of DA release. Furthermore, TRH also has a presynaptic regulatory role in modulating TIDA neuron activity through interactions with glutamatergic and GABAergic neurons [24]. For example, TRH increases both the frequency and amplitude of excitatory postsynaptic potentials, whereas it increases the frequency and decreases the amplitude of inhibitory postsynaptic potentials in TIDA neurons. Collectively, this demonstrates the pleiotropic mechanisms by which TRH neurons augment the excitability of TIDA neurons and, consequently, enhance the release of DA to suppress PRL secretion.

#### 2.5.2. Opioids

Both exogenous and endogenous opioids are well-recognized as stimulators of PRL release. Their regulatory influence on PRL secretion is indirectly mediated through TIDA neurons [64,65,66,67,68], with limited evidence to support the direct stimulation of lactotrophs by opiates for PRL release [69,70]. Thus, opioids directly inhibit TIDA neurons and decrease DA output [71,72,73]. The regulation of TIDA neurons by opioids involves a complex interplay of various receptor subtypes, each serving distinct physiological functions. In 1992, Manzanares et al. delved into the role of kappa opioid receptor-mediated regulation on TIDA neurons. Their findings underscore that the endogenous opioid dynorphin, acting through kappa receptors, inhibits DA output from TIDA neurons, consequently leading to increased PRL secretion [74]. This regulatory process is modulated by gonadal hormones like estradiol, hinting at sex differences in the basal activity of TIDA neurons through the tonic inhibitory regulation by dynorphin [41]. Loose et al. made significant contributions to our understanding of how opioids reshape the firing patterns of TIDA neurons, inducing inhibitory effects. Through the use of selective mu-receptor agonists, they observed a concentration-dependent decrease in the firing rate of ARC neurons, which could be reversed by naloxone [75]. Their subsequent study revealed that the decreased firing rate in ARC neurons induced by mu-receptor agonists was attributed to an increase in inwardly rectifying potassium conductance and the subsequent hyperpolarization of ARC neurons [76]. These effects occur in TIDA neurons identified immunocytochemically through the presence of TH [76]. These neurons became hyperpolarized through the induction of an outward current, and this outward current exhibited a reversal potential consistent with selective potassium conductance. Turning to another neuropeptide within the opioid family, Orphanin Nociceptin/orphanin FQ (N/OFQ), Wagner et al. explored its effects on ARC neurons in the hypothalamus in 1998. Their research unveiled an N/OFQ-induced hyperpolarization in various neuron types, including those containing beta-endorphin, tyrosine hydroxylase, and gonadotropin-releasing hormone. This hyperpolarization was dose-dependent and unaffected by naloxone, pointing to non-classical opioid receptor involvement. Alterations in extracellular potassium levels and the use of barium chloride confirmed the participation of G-protein-gated, inwardly rectifying potassium channels. These findings illuminate the role of OFQ in modulating TIDA neurons, through the activation of specific potassium channels, ultimately impacting PRL secretion [77]. These findings strongly suggest a direct postsynaptic inhibition of TIDA neurons by activation of several different opioid receptor subtypes and highlight that TIDA neurons are a physiologically relevant target of opioid peptides [10].

#### 2.5.3. Neurotensin (NT)

The majority of neurosecretory cells capable of synthesizing NT are predominantly located in the ARC [78]. These NT-synthesizing neurons have the capacity to exert direct or indirect influence on neurosecretory cells, including TIDA neurons and, thus, its tonic inhibition of PRL secretion [79]. To investigate the precise mechanisms through which NT affects TIDA neurons, an experiment was conducted by Pan and their team. In ovariectomized rats, they administered NT intracerebroventricularly and measured the levels of 3,4-DOPAC in the ME. The results revealed that NT stimulated DA secretion from tuberoinfundibular neurons. Simultaneously, there was a reduction in the plasma level of PRL. These findings suggest that the increase in DA release caused by NT leads to the suppression of pituitary hormone secretion [80]. Another experiment involving the administration of exogenous NT either intracerebroventricularly or intravenously to rats provided further insights. It was observed that central administration of NT decreased PRL secretion, while intravenous administration increased PRL secretion [79]. This suggests that NT regulates the DA–PRL axis through multiple pathways. Additionally, further research has provided evidence that NT can directly stimulate PRL secretion at the level of the anterior pituitary [81,82]. Consequently, NT may exert negative feedback regulation on the same cell population from which it is co-released with DA, thereby altering their concentrations in the hypothalamus and the anterior pituitary [83].

#### 2.5.4. Bombesin (BBS)

BBS is a 14-amino acid neurohormone polypeptide, derived initially from amphibians, with a wide range of physiological effects in the brain, lungs, and GI tract. Research has also discovered several peptides structurally related to BBS [84]. Two well-studied homologs are called neuromedin B and gastrin-releasing peptide (GRP) [85]. BBS regulates gastrointestinal hormone release and gastrointestinal motility [85]. BBS stimulating PRL secretion in cultured tumor cells such as meningioma-derived cells and pituitary tumor cells has been reported [86,87]. In 1991, Manzanares et al. reported that BBS directly influences dopaminergic neurons, albeit primarily in the hypothalamus. According to a study by Manzanares and colleagues, intraventricular injection of BBS resulted in a dose- and time-dependent increase in the activity of TIDA neurons projecting to the ME. This increased activity was associated with a decrease in PRL levels in the bloodstream. Importantly, these alterations in the activity of DA neurons were not observed in other brain regions such as the striatum, nucleus accumbens, or neural lobe of the pituitary [88]. Moreover, research from the same group shed light on sexual differences in the stimulatory effects of BBS on TIDA neurons. Central administration of BBS decreased PRL secretion in both sexes, but an increase in the activity of TIDA neurons was observed only in males. However, there is an increase in the activity of TIDA neurons that can be observed in female rats after ovariectomy. This indicates that gonadal hormones like estradiol play a role in influencing the responsiveness of TIDA neurons to BBS, highlighting the complex interplay between BBS and hormones in the regulation of TIDA neuronal activity and pituitary PRL secretion [42].

#### 2.5.5. Galanin (GAL)

In the case of GAL, many cells in the pituitary, including lactotrophs, somatotrophs, and thyrotrophs, have been found to contain immunoreactive GAL. Notably, estradiol stimulates GAL mRNA expression, while DA inhibits GAL secretion in the rat anterior pituitary [89]. Research into how GAL regulates TIDA neurons in the central nervous system has revealed interesting insights. It was found that centrally administered GAL can rapidly stimulate PRL secretion in both male and female rats without affecting the baseline activity of TIDA neurons. However, in rats of both sexes whose TIDA neuronal activity was stimulated by the administration of the DA antagonist haloperidol, GAL decreased the ratio of DOPAC to DA in the ME. This suggests that GAL can inhibit TIDA neuron activity when these neurons are activated. These results imply that the activation of PRL secretion by GAL is not mediated by changes in the tonic inhibition of TIDA neurons and that TIDA neurons are responsive to the inhibitory effects of GAL only under certain activated conditions [90].

### 2.6. Regulation by Lactation

Throughout lactation, dopaminergic inhibition of lactotrophs remains low, and TIDA neurons do not increase DA output in response to high plasma PRL levels [11]. It is evident that reducing DA output from TIDA neurons to maintain high prolactin levels during lactation involves multiple contributing factors, for example, enhanced synthesis of neuropeptide Y, enkephalins, and neurotensin in TIDA neurons during lactation has been detected by immunolabeling [83]. These neuromessengers may be co-released together with DA from the ME to regulate suckling-induced PRL secretion.

Suckling-induced reductions in TIDA neuronal activity are well-established. TH mRNA in TIDA neurons is suppressed during lactation, followed by a rebound after pup removal [17]. Suckling also reduces DA turnover in the ME [18]. High endogenous prolactin levels from suckling may activate TH activity in early lactation, but TIDA neurons become less responsive to PRL feedback by day 13 of lactation [16]. Research on lactating rats demonstrates that opiate peptides regulate PRL release in response to suckling. In one study, naloxone effectively suppresses suckling-induced PRL release in a dose-dependent manner [91], while another study showed that μ but not κ opioid receptor antagonists increase DOPA accumulation in the ME of lactating rats, demonstrating their impact on TIDA neuronal activity [92]. Additional evidence supporting opioid peptide-induced suppression of TIDA neurons during lactation comes from a study showing that beta-endorphin neurons play a key role in the suckling-induced inhibition of TIDA neurons, whereas naloxone leads to a significant increase in TH activity and a reduction in circulating PRL [93]. Interestingly, the expression of clock genes like PER1 is highly entrained to scheduled suckling in lactating female rabbits [94].

Suckling-induced oxytocin secretion or systemically increased oxytocin can stimulate PRL secretion through actions on lactotroph cells [95,96,97]. However, central administration of oxytocin causes a reduction in plasma PRL [95]. Research shows that oxytocin excites TIDA neurons in both lactating and non-lactating rats. Oxytocin induced a shift from rhythmic activity to tonic discharge by directly depolarizing TIDA neurons. The depolarization is mediated by a voltage-dependent inward current, consisting of a transient receptor potential-like conductance in the low-voltage range and a calcium-dependent component in the high-voltage range. This activation of TIDA neurons by oxytocin might serve as a feedforward inhibition of PRL release [98]. It is clear that the mechanisms underlying the duality in oxytocin’s effects on PRL secretion are complex and involve intricate interactions between various hormones and neural circuits in the hypothalamus.

**Figure 3:**
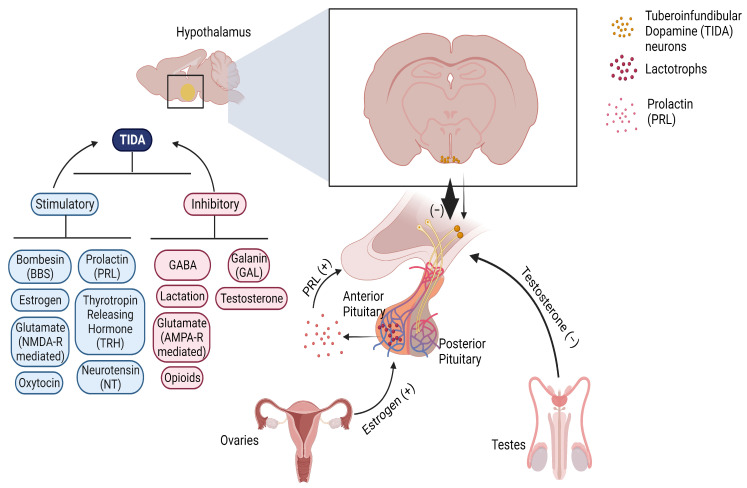
Schematic summarizing the function and regulation of TIDA neurons. TIDA neurons have cell bodies located in the arcuate nucleus of the mediobasal hypothalamus. Their axons terminate in the median eminence, and the released dopamine is carried via the hypophysial portal vasculature to the anterior pituitary where it tonically inhibits prolactin (PRL) secretion from lactotrophs. Estrogens like estradiol stimulate PRL synthesis and secretion, which then stimulates TIDA neurons to suppress further PRL secretion. By contrast, testosterone inhibits these neurons. TIDA neurons are also regulated by numerous excitatory and inhibitory small molecule and neuropeptide transmitters, indicated on the left-hand side of the figure.

Additionally, Romanò et al. investigated the changes in the electrical behavior of TIDA neurons during lactation, as membrane properties and discharge patterns influence dopamine release. TIDA neurons themselves do not undergo significant changes in their electrical properties, but dopamine secretion decreases in the ME during lactation. TIDA neurons remain electrically responsive to PRL stimulation, showing an acute increase in their firing rate. However, lactation was not found to alter significantly either the firing rate or pattern of TIDA neurons. These findings suggest a decoupling of electrical activity and dopamine secretion. This phenomenon may relate to the plasticity of TIDA neurons during lactation, with the lack of phosphorylation of TH partially contributing to the reduction in dopamine secretion [99]. Therefore, the role of firing patterns in the plasticity of TIDA neuron output remains an open question, highlighting the need for thoroughly investigating the unique features of TIDA neurons during lactation, particularly in different neuron subgroups. The complex regulatory interplay between TIDA neurons and the various factors involved is shown in Figure 3.

## 3. Concluding Remarks and Future Directions

A large amount of information has been gathered over the past several decades regarding the function and regulation of TIDA neurons. The transition from in vivo neurochemical estimates of TIDA neuronal activity to more sophisticated intracellular recording techniques has enabled the neuroscience community to probe deeper into the true cellular determinants of excitability. This transition has dispelled long-held notions that things like the suckling reflex and TRH increase PRL secretion by inhibiting the activity of TIDA neurons, when, in fact, both increase the excitability of these cells. Perhaps the increased TIDA neuronal excitability is part of a feedback mechanism aimed at curbing the suckling- and TRH-induced increases in PRL secretion. Future studies should endeavor to reconcile these disparities. Future considerations should also include investigating alternative downstream targets of TIDA neurons in addition to pituitary lactotrophs. Indeed, in recent years, it has come to light that TIDA neurons stimulate energy intake by inhibiting anorexigenic proopiomelanocortin neurons and exciting orexigenic neuropeptide Y neurons in the ARC [100]. More studies of this nature will undoubtedly enhance our understanding and appreciation of the truly diverse role TIDA neurons play in the inexorable link between reproduction and energy balance.
